# Fingerprint Approaches Coupled with Chemometrics to Discriminate Geographic Origin of Imported Salmon in China’s Consumer Market

**DOI:** 10.3390/foods10122986

**Published:** 2021-12-03

**Authors:** Xianshu Fu, Xuezhen Hong, Jinyan Liao, Qingge Ji, Chaofeng Li, Mingzhou Zhang, Zihong Ye, Xiaoping Yu

**Affiliations:** 1 Zhejiang Provincial Key Laboratory of Biometrology and Inspection & Quarantine, College of Life Science, China Jiliang University, Hangzhou 310018, China; fxs@cjlu.edu.cn (X.F.); QinggeJi1997@163.com (Q.J.); lcf609162059@163.com (C.L.); zhye@cjlu.edu.cn (Z.Y.); yxp@cjlu.edu.cn (X.Y.); 2 College of Quality & Safety Engineering, China Jiliang University, Hangzhou 310018, China; xzhong@cjlu.edu.cn; 3 Zhejiang Yuying College of Vocational Technology, Business and Trade Branch, Hangzhou 310018, China

**Keywords:** salmon, near-infrared (NIR), mineral element fingerprint (MEF), data preprocessing, principal component analysis (PCA), partial least squares (PLS)

## Abstract

Of the salmon sold in China’s consumer market, 92% was labelled as Norwegian salmon, but was in fact was mainly imported from Chile. The aim of this study was to establish an effective method for discriminating the geographic origin of imported salmon using two fingerprint approaches, Near-infrared (NIR) spectroscopy and mineral element fingerprint (MEF). In total, 80 salmon (40 from Norway and 40 from Chile) were tested, and data generated by NIR and MEF were analysed via various chemometrics. Four spectral preprocessing methods, including vector normalization (VN), Savitzky Golay (SG) smoothing, first derivative (FD) and second derivative (SD), were employed on the raw NIR data, and a partial least squares (PLS) model based on the FD + SG9 pretreatment could successfully differentiate Norwegian salmons from Chilean salmons, with a R^2^ value of 98.5%. Analysis of variance (ANOVA) and multiple comparative analysis were employed on the contents of 16 mineral elements including Pb, Fe, Cu, Zn, Al, Sr, Ni, As, Cr, V, Se, Mn, K, Ca, Na and Mg. The results showed that Fe, Zn, Al, Ni, As, Cr, V, Se, Ca and Na could be used as characteristic elements to discriminate the geographical origin of the imported salmon, and the discrimination rate of the linear discriminant analysis (LDA) model, trained on the above 10 elements, could reach up to 98.8%. The results demonstrate that both NIR and MEF could be effective tools for the rapid discrimination of geographic origin of imported salmon in China’s consumer market.

## 1. Introduction

Salmon, the scientific name of the Atlantic salmon (*Salmo salar*) [1], is a Salmonidae fish favoured worldwide for its high protein and vitamins [2,3], low fat and cholesterol [4,5], pleasurable taste and aroma [6,7], and putative health effects [8,9], especially by Chinese consumers. According to the Food and Agriculture Organization (FAO) [10], the global supply of salmon exceeded 2,540,000 tonnes in 2019, with the figure steadily increased to 2,680,000 tonnes in 2020, by the same, the outputs of salmon from Norway and Chile were 1,400,000 more tonnes and 700,000 fewer tonnes, respectively. Regarding salmon, a slower growth was proved for aquaculture production in Norway (from 380,000 to 1,200,000 tonnes) compared to that of Chile’s aquaculture of salmon (from 150,000 to 600,000 tonnes); indeed, Norway and Chile were the two largest exporters of salmon in the world. Norwegian salmon were more popular due to their low presence of antibiotics (astaxanthin feeding), strong aroma, bright colour, unique and delicious taste, which also made Norwegian salmon more expensive than Chile’s. Substitution and high-priced fish replaced by cheaper alternatives [11,12,13], is a commonplace problem, which can occur at every stage of the food chain before sale. According to statistics, more than a third of seafood imported into the United States was reported mislabelled [14,15], and the label error rate all over the world was about 3.4 to 30% [16,17,18]. The General Administration of Customs P.R. China conducted surveys in supermarkets and wholesale markets across the country from 2015 to 2018, and the result showed that 92% of salmon sold in China’s consumer market was labelled as salmon imported from Norway but was in fact mainly from Chile. Therefore, it is urgent to establish effective methods for authenticity of imported salmon in China, which could also provide a technical reference for the establishment of market supervision system of imported salmon.

Traditional research on the adulteration of salmon has mainly focused on the following three areas: species identification, production mode (wild or farmed) [19,20,21] and origin identification. Many studies have identified species using molecular markers [22,23], DNA profiling [24,25] and droplet digital PCR (ddPCR) [26], all of which could achieve accurate single or multiple species identification results. There are also some methodologies utilised to determine the origin of seafood, among which element fingerprint (EF) [27], stable isotope analysis (SIA) [28] and fatty acid profiling were the most familiar used detection methods. For the EF approach, two similar devices were commonly employed for detection, namely, Inductively Coupled Plasma-Mass Spectrometry (ICP-MS) [29,30] and Inductively Coupled Plasma-Optical Emission Spectrometry (ICP-OES) [31,32]. ICP-MS/OES can be applied to distinguish the geographic provenances of non-identified seafood by the naked eye [33]. Cubadda et al. [34] were able to clearly distinguish the Mediterranean mussel sites of three adjacent fisheries via ICP-MS but failed to identify young mussels collected from habitats as far as 11 km away. Similarly, Costas-Rodriguez et al. [35] combined ICP-MS and linear discriminant analysis (LDA) to predict and distinguish five gathering locations of *M. galloprovincialis.* Li et al. [36] used ICP-OES to detect 20 elements variables of Pacific white shrimp and established the geographical origin-discriminant model by combining PCA and other chemometrics methods. However, ICP-MS/OES has a few drawbacks, such as complicated sample pretreatment operation, expensive instruments and easy tampered with element content [37,38]. Zhang et al. [39] determined δ^13^C and δ^15^N values of 575 scallop samples from 7 habitats around China by SIA method, and analysed the above isotopes combination with fisher linear discriminant analysis (LDA). LDA results showed that the classification accuracy of scallop origin prediction reached 92%, demonstrating that the stable carbon and nitrogen isotopic compositions of scallops from different habitats, seasons, and species were significantly different. Grazina et al. [40] used a gas chromatography-flame ionization detector (GC-FID) to analyse the fatty acid contents of wild-caught and farmed salmon from Canada, Chile and Norway (n = 26,25,24,25, 26 wild from Canada and the rest from aquaculture plants of three countries, respectively), and combined with principal component analysis (PCA) for provenance analysis. The results showed that PCA could not clearly distinguish the geographical origin of salmon due to the overlapping of aquiculture salmon samples between Canada and Chile. Nevertheless, using 17 fatty acid contents in the models, six machine learning classifiers, ANN, Naïve Bayes, Random Forest, SVM and kNN, all achieved 100% classification accuracy on the test data set [40]. Tian et al. [41] combined an XGBoost algorithm and ICP/MS to screen 10 elements from 30 elements, before distinguishing Huanghua fruit from other research regions, with an overall authenticity accuracy of 95%.

However, these aforementioned research works only concerned one single instrument, while many research have already demonstrated better performance by joint use of two analytical approaches. The aim of this study was to establish an effective method for discriminating the geographic origin of imported salmon using two fingerprint approaches, Near-infrared (NIR) spectroscopy and mineral element fingerprint (MEF).

## 2. Materials and Methods

### 2.1. Salmon Samples, Reagents, Instruments and Equipments

**Figure 1:**
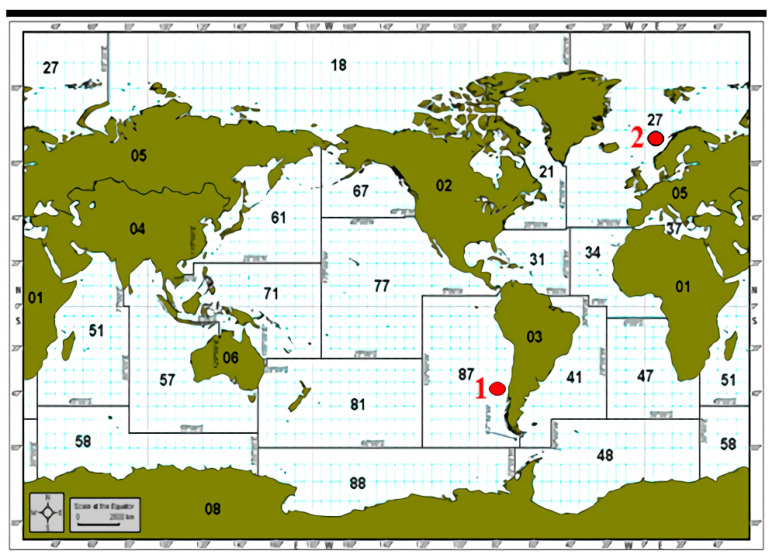
Diagram of collection area of imported salmon in Chile (1) and Norway (2).

All salmon (40 from Norway and 40 from Chile) were imported by Zhejiang Beiji Product Aquatic Products Co., LTD. As shown in Figure 1, the Chilean salmon were farmed in Location 1 and the Norwegian salmon were farmed in Location 2. In order to ensure the uniformity of research materials, all salmon covered with skin were stored at −20 °C refrigerator before measuring.

**Figure 2:**
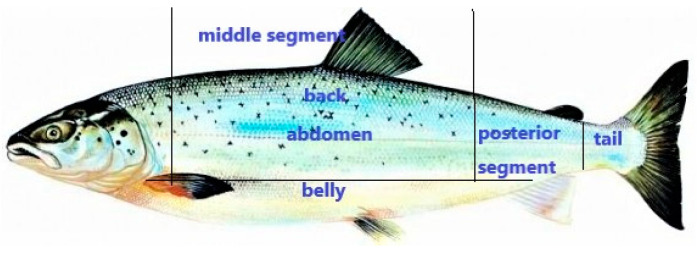
Body diagram of a salmon.

As observed in Figure 2, the flesh of salmon can mainly be divided into five parts: tail, posterior segment, belly, abdomen and back. Fat content of the five parts listed in descending orders are belly, abdomen, back, posterior segment and tail. Correspondingly, the middle segment, especially the belly part of a salmon, has the best taste and is thus the most expensive.

Concentrated nitric acid (65%, analytically pure), H_2_O_2_ (30%, analytically pure) and hydrochloric acid (37%, analytical pure) were bought from China National Pharmaceutical Group Co., Ltd. (Shanghai, China). Mineral element standard samples (1 mg mL^−1^ per element), such as Ag (I), Cd (II), Pb (II), Fe (III), Cu (II), Zn (II), Al (III), Sr (I), Ni (II), As (III), Cr (III), V (V), Se (VI), Mn (II), K (I), Ca (II), Na (I) and Mg (II), were purchased from the National Public Service Platform for Standards Information (Beijing, China).

### 2.2. Near-Infrared (NIR) Spectroscopy

#### 2.2.1. Sample Preparing and Detection

Near-infrared (NIR) spectroscopy was performed to generate spectral data of the salmon samples. For each salmon, five samples were collected from its tail, posterior segment, belly, abdomen and back, respectively. Each sample, weighing 20 g, was cut into cubes with an edge length of 25–30 cm. Thus, in total, there were 400 salmon samples (5 parts from of each 80 salmon).

NIR spectroscopy employed in this research was Tensor37 NIR spectrometer (Bruker Analytical Instrument Group Co., Ltd., Karlsruhe, Germany). Working conditions were set at room temperature of 25 °C, humidity < 70%, 3 nm of diaphragm and 10 kHz of scanning frequency. The scanning was done on the fresh, and average values of 64 scanning spectra ranging from 4000 cm^−1^ to 12,000 cm^−1^ were considered as the raw NIR data. Since scanning interval was set at 3.856 cm^−1^, the output data contained 2074 spectral points. Thus, the size of the raw dataset (Norway and Chile) was 400 samples × 2074 variables.

All the spectra were analysed by a highly sensitive indium gallium arsenide (InGaAs) detector, which could provide a linear response across all wavebands for better accuracy and reproducibility. An internal background scanning was measured with gold background before detection as the reference.

#### 2.2.2. Spectral Data Preprocessing

In order to enhance the performance of discrimination modelling, different data pretreatment methods were introduced. The purpose of pretreatment was to eliminate the influence of external factors (such as sample background, noise, stray light, etc.) on the spectral data. Savitzky Golay (SG) [42] smoothing was usually applied to improve the signal-to-noise ratio (SNR) and to decrease the noise of the original spectra. In this research, SG9 smoothing (three filter windows with a filter width of nine points) was employed to boost NIR resolution and lessen background and baseline. Vector normalization (VN) [43] was taken to reduce the difference between multiple measurements of the same variety and weaken the spectral difference caused by small optical path difference. First derivation (FD) and second derivation (SD) were used to remove the spectrographic variations caused by variations of sample size and lost optical path and increase the resolution and sensitivity.

### 2.3. Mineral Element Fingerprint (MEF)

#### 2.3.1. Sample Preparing and Pretreatment

As shown in Figure 2, belly, abdomen and back formed the middle portion of salmon, which were the main parts of the salmon flesh. The weight of the salmon was usually 4 to 6 kg, even reaching 10 kg. It was extremely difficult to homogenise a whole salmon, so we randomly selected the middle portion as the representative sample. The middle portion of every salmon sample was randomly chosen, cutting into small pieces with a sterile knife, and drying at 70 °C in a steady temperature oven until the weight of selected salmon was constant. The baked salmon was cooled to room temperature in a dryer, and then ground to powder with a ceramic mortar and pestle. An electronic analytical balance was used to accurately weigh 0.3000 g of salmon powder and put it into a dry digestion tube; 6.00 mL concentrated nitric acid and 3.00 mL hydrochloric acid were successively added into the digestion tube before being enclosed into a Mars microwave digestion machine (CEM Co., Ltd., Matthews, NC, USA).

The working procedures of Mars microwave digestion instrument in this research were as follows: (1) to gradually increase the power to 1600 W and the temperature to 120 °C within 5 min, and hold for 3 min; (2) to heat up from 120 to 185 °C for no more than 6 min and keep for 10 min; (3) to rise from 185 to 240 °C in 5 min and maintain at this temperature for 40 min; and (4) to cool rapidly to room temperature in less than 20 min. After digestion and cooling, approximately 1 mL of clear and transparent solution was obtained. The solution was transferred to a 50 mL empty volumetric flask, and the digestive tube was washed with ultra-pure water (18.2 M ω cm) three times, which was also shifted from a volumetric flask to a centrifuge tube. Finally, the digestion solution was fixed to 50 mL and stored in the refrigerator for later use.

#### 2.3.2. MEF Detection

MEF analysis of 18 above elements were, respectively, employed and obtained from Elan Drc-E ICP-MS (Perkin Elmer Co., Ltd., Waltham, MA, USA) and Optima 8000 ICP-OES (Perkin Elmer). The contents of 13 elements including Ag (Ⅰ), Cd (II), Pb (II), Fe (III), Cu (II), Zn (II), Al (III), Sr (Ⅰ), Ni (II), As (III), Cr (III), V (Ⅴ) and Se (VI) were determined by ICP-MS, and the remaining five elements (i.e., Mn (II), K (Ⅰ), Ca (II), Na (Ⅰ) and Mg (II)) were detected using ICP-OES. ICP-MS/OES was calibrated with a blank control that did not contain samples. The measurements were repeated three times for each sample, and each value calculated was the average of the three measurements. The comparison of information regarding the optimised working conditions and parameters for ICP-MS and ICP-OES is shown in Table 1.

### 2.4. Data Analysis Approaches

Principal component analysis (PCA) [44,45] are often applied for dimension reduction. High-dimensional data could be projected into a point in two-dimensional or three-dimensional space after being processed, which could be analysed intuitively by naked eyes. In this study, PCA method was used for visualisation of NIR spectra. After data centralization, singular value decomposition (SVD) was carried out, then a set of biorthogonal vectors was obtained after SVD. Among them, the more advanced data had a larger variance on this vector, and thus the first three vectors were taken as a cartesian coordinate system. The NIR data were projected on this vector, respectively, and the three values obtained were the principal component distribution of the sample data in this space. The more similar the data was, the closer the principal component distribution was. The principle was to project the original sample data to a new coordinate system, in which only the largest two-dimensional or three-dimensional coordinates corresponding to the largest linear unrelated eigenvalue of the original sample were selected. In this way, the NIR data were clustered and classified.

Partial least squares (PLS) [46,47] was the most common stoichiometric analysis method, which also recently served as an alternative to soft independent modelling of class analogy (SIMCA) [48,49]. PLS was carried out as a regression of a dummy response variable y on the NIR spectral data X for solving multivariate quality control problem. The PLS model was based on the following special PLS regression according to the following output Formula (1)

(1) $$1={\mathrm{Xb}}_{\mathrm{PLS}}+e$$

where X is the measurement matrix of the target class (n × p, n and p represent the numbers of samples and features of model, respectively); 1, b_PLS_, and e were denoted as the response vector (n × 1), the PLS regression coefficient and the model error, respectively. In this research, the corresponding y is set at 1 for Norwegian salmon and −1 for Chilean salmon. A new sample was classified as Norwegian salmon with a predicted response greater than 0, and similarly, a predicted response less than 0 was classified as Chilean salmon. The model complexity of PLS models was estimated by Monte Carlo Cross-Validation (MCCV) [50,51,52].

During modelling, 70% of the dataset from each group (Norway and Chile) was randomly chosen as the training set, and the rest (30%) was considered as the testing set. This random-split process was repeated 50 times, and the average training and testing results were recorded. Leave-one-out (LOO) technique was employed for cross-validation.

### 2.5. Other Approaches Employed and Software Implemented

One-way analysis of variance (ANOVA) was performed to determine if there were differences among different groups, and Tukey’s multiple comparison was performed to separate the means at *p* < 0.05.

All data analysis procedures were performed using Python 3, mostly the scikit-learn tool, which is a simple and efficient tool for data mining and data analysis.

## 3. Results and Discussion

### 3.1. Near-Infrared (NIR) Spectrum Analysis

The results of NIR spectra were output and saved in the form of data point table. Previous research has indicated that modelling based on a full-band spectrum is better than that established by partial band [53,54]. Due to the serious overlap of different spectra at the same wave number, it was difficult to perfectly complete the tracing model from a few characteristic bands. Therefore, the whole band spectral information was chosen to ensure the accuracy of the model for establishing the traceability model in this study.

**Figure 3:**
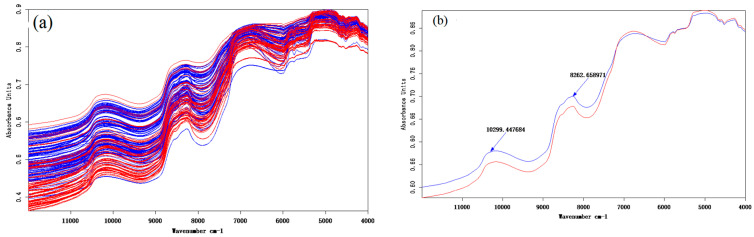
Original (**a**) and mean (**b**) spectrogram of Norwegian (blue) and Chilean (red) salmon.

The original (a) and mean (b) spectra of Norwegian (blue) and Chilean (red) salmon are presented in Figure 3, where there are different peaks at 10,299 cm^−1^, 8262 cm^−1^, 6869 cm^−1^, 5044 cm^−1^ and 4249 cm^−1^. The difference of wave peak between 10,299 cm^−1^ and 8262 cm^−1^ is remarkable, and there is an obvious absorption peak. Differences in wave peaks can only indicate differences in functional groups of organic compounds in salmon samples, and specific traceability research needs to be combined with stoichiometric methods for further accurate determination.

**Figure 4:**
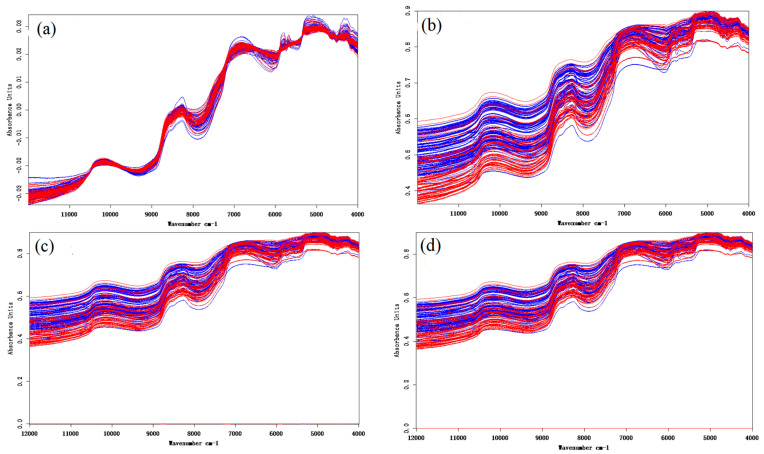
Pretreatment approaches for Norwegian (blue) and Chilean (red) salmon spectra by VN (**a**), SG9 (**b**), FD in combination with SG9 (**c**) and SD combined with SG9 (**d**).

Pretreatment approaches for Norwegian (blue) and Chilean (red) salmon spectra by VN (a), SG9 (b), FD in combination with SG9 (c) and SD combined with SG9 (d) are presented in Figure 4. Observed from Figure 4a, VN can only slightly reduce the difference between Norwegian salmon and that of Chile, indicating that the effect of VN is not significant. Observed from Figure 4b–d, these three pretreatments showed similar pattern as they all involved the SG9 pretreatments. In general, it is hard to tell which pretreatment approach is the best. The actual impacts of spectral pretreatment should be estimated by subsequent classification performance.

### 3.2. Principal Component Analysis (PCA)

**Figure 5:**
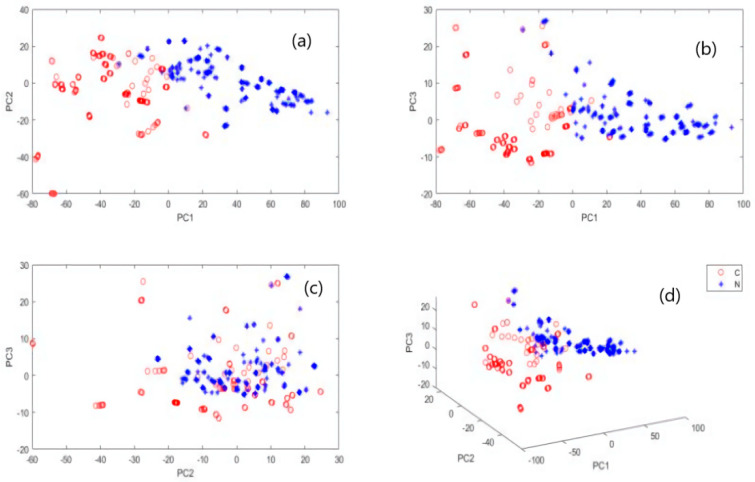
Distribution space of Norwegian (blue) and Chilean (red) salmon based on PC1-PC2 (**a**), PC1-PC3 (**b**), PC2-PC3 (**c**) and PC1-PC2-PC3 (**d**).

PCA visualisation of the raw dataset is shown in Figure 5, where the first three principal components of the cumulative variance contribution rate are 98%. As observed in Figure 5, the Norwegian and Chilean salmon could be visually separated, demonstrating the feasibility of using NIR spectroscopy to differentiate Norwegian and Chilean salmon.

### 3.3. Partial Least Square (PLS) Discrimination Model

PLS modelling based on different pretreatment approaches are presented in Table 2, where the root-mean-square error (RMSE) and correlation coefficient (R^2^) are employed to evaluate prediction performances.

As presented in Table 2, RMSE and R^2^ obtained by different spectra pretreatment methods were significantly different (*p* < 0.05), and all four approaches outperformed the control group (original spectra), demonstrating that spectral preprocessing could effectively promote the performance of PLS model. The PLS model based on FD in combination with SG9 has the highest R^2^ (0.983), indicating the FD in combination with SG9 (FD + SG9) approach is the best scheme for salmon discrimination model.

**Figure 6:**
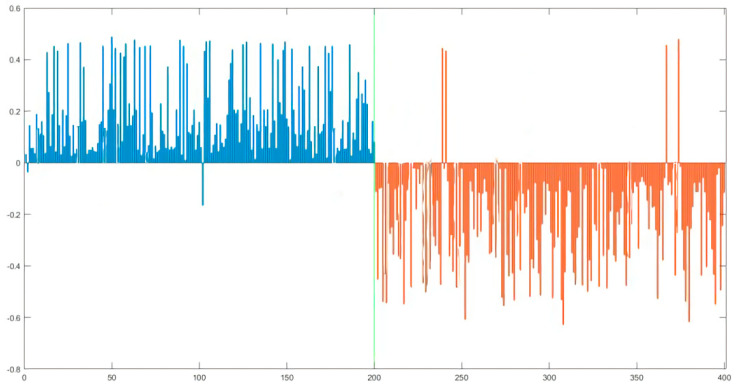
Prediction results of PLS model of Norwegian (blue) and Chilean (red) salmon based on FD in combination with SG9.

The result of PLS, based on the best scheme, is presented in Figure 5, where the predicted response of blue sample being greater than 0 indicates that the Norwegian sample was correctly classified. As observed in Figure 6, two Norwegian samples and four Chilean samples were misjudged. The overall discriminant rate of PLS model is 98.5%, meaning that the discriminant accuracy of PLS is slightly better than that of PCA.

### 3.4. Mineral Element Fingerprint (MEF) Analysis

#### 3.4.1. Differences in the Content of Mineral Element between Norwegian and Chilean Salmon

According to ICP-MS and ICP-OES, the contents of Cd and Ag in Norwegian and Chilean salmon were lower than the detectable value, so they were not analysed. Multiple comparative analysis of the contents of 16 mineral elements including Pb, Fe, Cu, Zn, Al, Sr, Ni, As, Cr, V, Se, Mn, K, Ca, Na and Mg, was conducted, and the mean values and standard deviation of mineral contents in Norwegian and Chilean salmon are shown in Table 3.

According to ICP-MS, there were significant differences (*p* < 0.05) of Fe, Zn, Al, Ni, As, Cr, V, Se, Ca and Na between Norwegian and Chilean salmon, while there were no significant differences (*p* > 0.05) in Pb, Mn, Cu, Sr, K and Mg. The mineral element content in fish is influenced by many factors, such as feed level, age, sex, mineral composition in water, fishing season and so on. Therefore, the mineral element content in salmon in the same area also varied greatly. The difference in mineral element content was not enough to be used as direct evidence to judge the regional difference, and the follow-up PCA was needed to establish the tracing mode.

#### 3.4.2. PCA of Mineral Elements in Norwegian and Chilean Salmons

**Figure 7:**
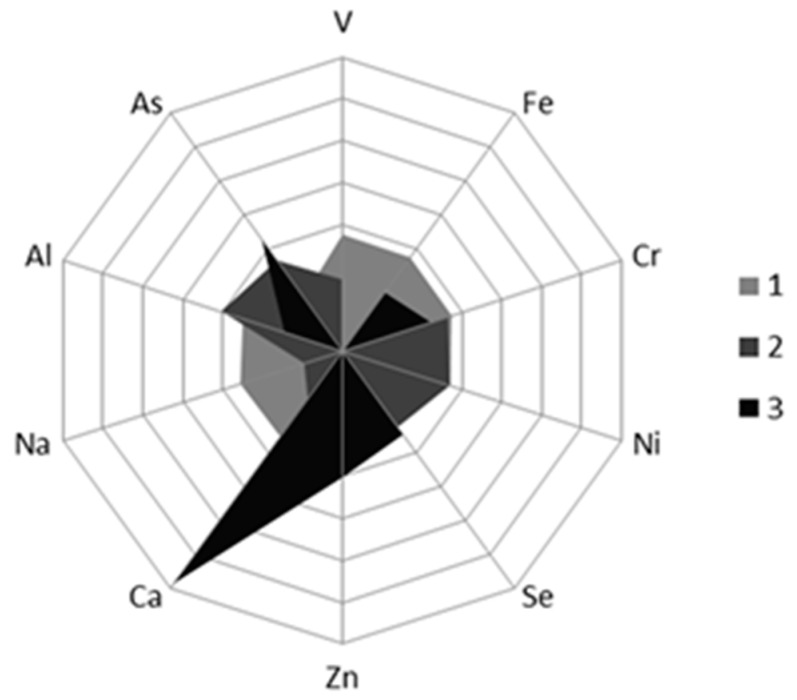
Eigenvector radar diagram of three principal components.

PCA was conducted and the first three PCs with cumulative variance contribution rate of 76.8% were extracted. Eigenvector radar diagram of the first three PCs was shown in Figure 7. As seen from Figure 7, the following conclusions can be drawn as follows: (1) the first principal component mainly integrated the content information of six elements, such as V, Fe, Cr, Ni, Zn and Na, (2) the second principal component primarily reflected the content information of Al, and (3) the third principal component principally incorporated the content information of three elements including Se, Ca and As.

**Figure 8:**
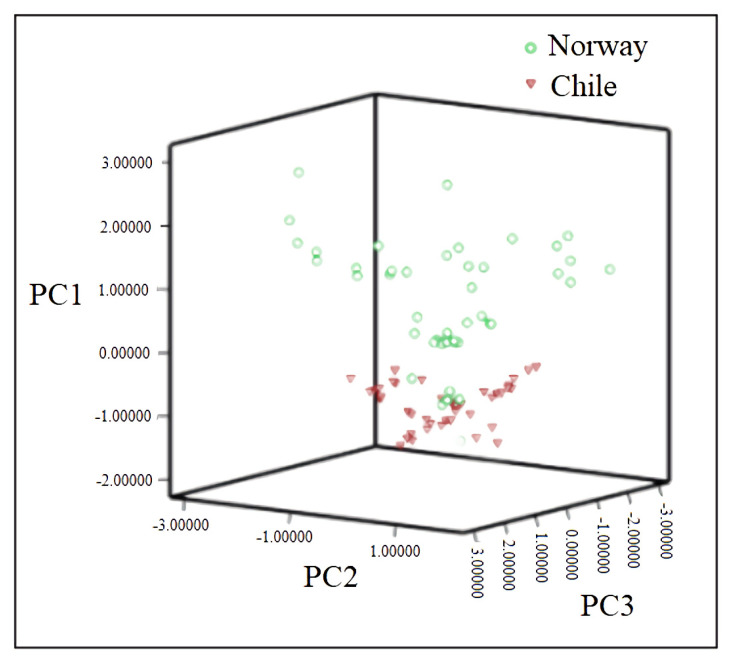
Score diagram of three principal components for Norwegian and Chilean salmon.

As presented in Figure 8, the Norwegian group (green) has a higher PC1 score and is thus distributed on the upper side of the 3D PCA plot, with only five samples overlapping with the Chilean group. This should be a strong proof that discrimination based on the mineral element content is possible.

#### 3.4.3. Linear Discriminant Analysis (LDA) of Mineral Elements

LDA was performed on 10 mineral elements with significant regional differences in Fe, Zn, Al, Ni, As, Cr, V, Se, Ca and Na. Fisher function and cross test were used for origin-discriminant analysis of salmon. During LDA modelling, LOO cross-validation was employed for calibration, and the corresponding equations of the best LDA model were as follows:

Y_N_ = −84.010 − 20.323V − 1.290Ni − 0.721Ca − 0.089Cr + 0.184Fe + 0.193Al + 0.600Zn + 1.555Na + 4.925As +17.449Se (2)

Y_C_ = − 138.722 − 24.471V − 0.893Cr − 0.668Ni − 0.429Ca + 0.157Al + 0.194Fe + 0.873Zn + 0.962Na + 4.283As + 28.240Se (3)

where Y_N_ and Y_C_ represented the corresponding equation for Norway salmon and that of Chile salmon, respectively. The above discriminant model was used to classify and analyse the collected samples. In the analysis of geographic origin of imported salmon, only one Norwegian salmon was wrongly estimated as Chilean salmon among the total of 80 samples, revealing that the overall provenance discriminant accuracy rate of Norwegian and Chilean salmon was 98.8%. The cross-validation result was the same as the above geographic origin result, i.e., 98.8%. Overall, this is a pivotal time for imported salmon provenance research, as MFF based on PCA and LDA are indicated valid and currently in booming.

## 4. Conclusions

In this research, NIR and MEF, combined with chemometric approaches, were employed to discriminate salmons imported from two different countries—Chile and Norway. The absorption peaks of NIR response to salmon samples at 10,299 cm^−1^ and 8262 cm^−1^ indicate that the difference between Chilean and Norwegian salmon spectra might be related to protein, water and other chemical components. The PLS model based on FD + SG9 pretreatment of the NIR spectra could successfully differentiate Norwegian salmons from Chilean salmons, with an R^2^ value of 98.5%. Regarding the MEF technique, all the 10 characteristic elements presented significant differences between Norwegian and Chilean salmons, and recognition rate of LDA based on the above 10 elements could reach up to 98.8%.

While these results are promising, further research is needed, as salmon collected in different seasons might have different levels of protein, and thus may generate slightly different NIR and MEF data. To make salmon origin discrimination models more robust and reliable, data updating is required. Inspired by this research, our next plan is to adjust these discrimination models (with the possibility to also explore more cutting-edge supervised techniques) for salmon collected in different seasons.

In addition, much research has indicated that simultaneous utilisation of two instruments might result in a better performance than individual utilisation when proper data fusion approaches are used. Thus, in subsequent research, we also plan to explore in studies if using perceptual knowledge from both NIR and MEF would increase the extent of information regarding salmon, or on the contrary, if they will they lead to data redundancy.

## Figures and Tables

**Table 1:** Optimized conditions and parameters of ICP-MS/OES.

Parameter	ICP-MS	ICP-OES
	Setting Value	Setting Value
Radio-frequency power	1300 W	1300 W
Scan times	100 times	100 times
scan patterns	Peak height	Peak height
Dwell time	10 ms	10 ms
Acquisition time	20 s	20 s
Sample uptake rate	1 mL/min	1.5 mL/min
Plasma gas flow	13 L/min	15 L/min
Coolant gas flow	15 L/min	12 L/min
Auxiliary gas flow	1.2 L/min	0.2 L/min
Atomizer gas flow	0.87 L/min	0.55 L/min

**Table 2:** RMSE and R^2^ of the discrimination model based on different pretreatment methods.

Pretreatment Methods	RMSE	R^2^
Original Spectra	0.198	0.879
VN	0.173	0.968
SG9	0.167	0.974
FD + SG9	0.159	0.983
SD + SG9	0.163	0.976

**Table 3:** Mineral Element Content of salmons from Norway and Chile.

Element	Index	Norway	Chile	Significant Difference
Pb (ppb)	content	0.61 ± 0.14	0.53 ± 0.28	No
	variable coefficient (%)	22.4	53.5	
Fe (ppb)	content	115 ± 35.9	102 ± 24.7	Yes
	variable coefficient (%)	31.3	24.1	
Mn (ppb)	content	1.29 ± 2.16	0.71 ± 1.16	No
	variable coefficient (%)	168	164	
Cu (ppb)	content	6.10 ± 0.99	6.40 ± 1.19	No
	variable coefficient (%)	16.2	18.4	
Zn (ppb)	content	63.9 ± 11.7	57.9 ± 7.65	Yes
	variable coefficient (%)	18.4	13.2	
Al (ppb)	content	44.6 ± 20.1	30.9 ± 17.0	Yes
	variable coefficient (%)	45.2	55.1	
Sr (ppb)	content	0.21 ± 0.79	0.03 ± 0.02	No
	variable coefficient (%)	369	92.3	
Ni (ppb)	content	2.39 ± 1.28	1.28 ± 0.83	Yes
	variable coefficient (%)	53.6	64.8	
As (ppb)	content	3.07 ± 1.03	3.99 ± 0.70	Yes
	variable coefficient (%)	33.6	17.5	
Cr (ppb)	content	16.3 ± 3.23	12.6 ± 2.90	Yes
	variable coefficient (%)	19.9	23.1	
V (ppb)	content	0.35 ± 0.24	0.09 ± 0.14	Yes
	variable coefficient (%)	70.2	167	
Se (ppb)	content	4.18 ± 0.63	6.82 ± 0.49	Yes
	variable coefficient (%)	15.4	7.13	
K (ppm)	content	65.7 ± 7.78	65.9 ± 6.37	No
	variable coefficient (%)	11.8	9.66	
Ca (ppm)	content	2.28 ± 2.20	1.14 ± 0.52	Yes
	variable coefficient (%)	96.8	45.6	
Na (ppm)	content	13.8 ± 4.05	9.26 ± 3.11	Yes
	variable coefficient (%)	29.3	33.6	
Mg (ppm)	content	4.79 ± 0.77	5.00 ± 0.37	No
	variable coefficient (%)	16.1	7.41	
